# Molecular pathways in experimental glaucoma models

**DOI:** 10.3389/fnins.2024.1363170

**Published:** 2024-03-18

**Authors:** Klaudia Bugara, Anna Pacwa, Adrian Smedowski

**Affiliations:** ^1^Department of Physiology, Faculty of Medical Sciences in Katowice, Medical University of Silesia, Katowice, Poland; ^2^GlaucoTech Co., Katowice, Poland; ^3^Department of Ophthalmology, Faculty of Medical Sciences in Katowice, Medical University of Silesia, Katowice, Poland; ^4^Department of Ophthalmology, Professor K. Gibinski University Clinical Center, Medical University of Silesia, Katowice, Poland

**Keywords:** glaucoma, glaucoma models, apoptosis, retinal ganglion cell, neuroprotection

## Abstract

Glaucoma is a complex and progressive disease that primarily affects the optic nerve axons, leading to irreversible vision loss. Although the exact molecular mechanisms underlying glaucoma pathogenesis are not fully understood, it is believed that except increased intraocular pressure, a combination of genetic and environmental factors play a role in the development of the disease. Animal models have been widely used in the study of glaucoma, allowing researchers to better understand the underlying mechanisms of the disease and test potential treatments. Several molecular pathways have been implicated in the pathogenesis of glaucoma, including oxidative stress, inflammation, and excitotoxic-induced neurodegeneration. This review summarizes the most important knowledge about molecular mechanisms involved in the glaucoma development. Although much research has been done to better understand the molecular mechanisms underlying this disease, there is still much to be learned to develop effective treatments and prevent vision loss in those affected by glaucoma.

## Introduction

Glaucoma, a silent thief of vision, affects millions of people worldwide (Tham et al., 2014). It is a complex eye condition characterized by progressive damage to the optic nerve axons, which are responsible for transmitting visual information from the retina to the brain (Osborne, 2008; Weinreb et al., 2014). The onset of glaucoma can be gradual and often has no symptoms until it has caused significant vision impairment, making early detection and treatment crucial for preventing blindness (Wagner et al., 2022). There are various types of glaucoma, including primary open-angle glaucoma, angle-closure glaucoma, and secondary glaucoma, each with unique causes and risk factors (Davis et al., 2016; XXX, 2017; Nakazawa and Fukuchi, 2020). Risk factors for glaucoma include age, family history, elevated intraocular pressure (IOP), high refractive errors, this cornea, and certain medical conditions, such as diabetes and high blood pressure (Chauhan et al., 2010; Peters et al., 2013). Diagnosis of glaucoma involves a comprehensive eye examination, including measuring IOP, performing visual field tests, and evaluating the optic nerve for signs of damage. Treatment for glaucoma may include prescription eye drops, laser therapy, or surgery, depending on the type and severity of the condition. It is imperative for individuals to understand the causes, symptoms, and risk factors of glaucoma to take steps to protect their eye health and preserve their vision. Regular eye exams, especially for those at high risk for developing glaucoma, are critical for early detection and effective treatment of this condition.

Many glaucoma patients do not exhibit elevated IOP (Mallick et al., 2016). Consequently, conventional treatments primarily focused on reducing IOP may not effectively manage all forms of glaucoma. This highlights the heterogeneous nature of the disease, with multiple underlying mechanisms contributing to its development and progression. For individuals with normal tension glaucoma (NTG) or other forms where IOP remains within the normal range, alternative therapeutic approaches beyond IOP reduction are necessary to address the diverse etiology of the condition. Such approaches may include targeting neuroprotective pathways, addressing vascular dysfunction, mitigating neuroinflammation, or enhancing cellular resilience against oxidative stress. By recognizing the variability in IOP levels among glaucoma patients and acknowledging the limitations of IOP-centric treatments, there is an opportunity to develop more tailored and effective management strategies.

One of the key signaling pathways involved in glaucoma is the apoptotic pathway (Levkovitch-Verbin, 2015). Apoptosis, or programmed cell death, has been shown to play a role in the death of retinal ganglion cells (RGCs), the nerve cells responsible for transmitting visual information to the brain, in glaucoma. Studies have shown that elevated IOP and oxidative stress, two key factors in the development of glaucoma, can activate apoptotic signaling pathways, leading to the death of RGCs (Mochizuki et al., 2012; d’Azy et al., 2016). Another signaling pathway involved in glaucoma is the oxidative stress pathway (Levkovitch-Verbin et al., 2000; Ferreira et al., 2004; Ozdemir et al., 2009; Ghanem et al., 2010; Erdurmuş et al., 2011; Majsterek et al., 2011; Bagnis et al., 2012; Himori et al., 2013; Goyal et al., 2014; Lin and Kuang, 2014; d’Azy et al., 2016; Naguib et al., 2021; Sanz-Morello et al., 2021). Oxidative stress, a state of imbalance between pro-oxidant and antioxidant molecules in the eye, can induce the activation of signaling pathways that lead to cellular damage and death. In glaucoma, oxidative stress has been shown to induce the death of RGCs, and antioxidant therapy has been shown to be protective in some animal models of the disease (Ozdemir et al., 2009; Himori et al., 2013; Lin and Kuang, 2014; Chen L. et al., 2015; Hines-Beard et al., 2016; Wenmin et al., 2016). Inflammation is another signaling pathway that is involved in the development of glaucoma (Wei et al., 2019; Wong and Benowitz, 2022). Inflammation in the eye can activate signaling pathways that lead to the death of RGCs and the progression of the disease. Studies have shown that elevated IOP and oxidative stress can activate pro-inflammatory signaling pathways in glaucoma, leading to the release of cytokines and other signaling molecules that contribute to cellular damage and death (Yang et al., 2011; Wilson et al., 2015).

The molecular mechanisms underlying the development and progression of glaucoma are not fully understood, but recent research has shed light on some of the key processes involved. In this review, we will delve deeper into the molecular mechanisms of glaucoma. We will also examine the role of oxidative stress, inflammation, and neurodegeneration in the pathogenesis of the disease, and how they contribute to damage to the RGCs. Furthermore, we will review recent advances in molecular biology and genetics that have shed light on the genetic risk factors and molecular pathways that underlie the development of glaucoma. Understanding the molecular mechanisms of glaucoma is crucial for the development of effective treatments and preventative strategies, and ongoing research is likely to lead to new insights and therapeutic approaches for this debilitating disease (Sulak et al., 2024).

## Animal models of glaucoma

**Figure 1:**
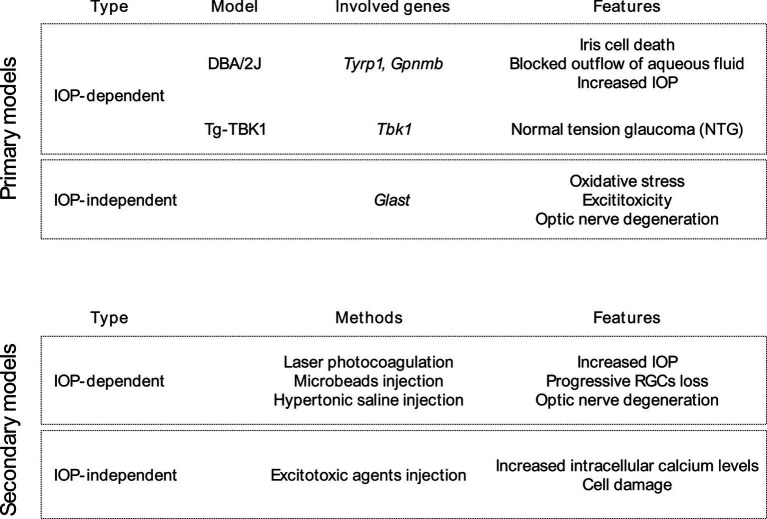
Primary and secondary glaucoma animal models with consideration of IOP-impact.

Like many other neurological conditions, comprehending the pathophysiology of glaucoma presents challenges in both laboratory settings (*in vitro*) and living organisms (*in vivo*). As a result, investigators depend on diverse animal models to examine the disease, with many of these models accurately mimicking crucial facets of glaucoma. Rodents are presently the predominant choice among animals for conducting glaucoma research (Kimura et al., 2020; Pang and Clark, 2020). These animals are appealing for several reasons, including their suitability for experimental manipulation (including genetic manipulation), short lifespan, cost-effectiveness in terms of maintenance, and a relatively similar eye structure and physiology to humans. There are many ways to induce glaucoma in animal models. There are primary models in which glaucoma has been induced by genetic manipulation (Harada et al., 2019), both IOP-dependent and IOP-independent, and secondary models, involving the manual induction of RGCs death, both by increased IOP or non-IOP related mechanisms (Figure 1).

The DBA/2 J mouse strain stands out as the most commonly used and thoroughly studied primary genetic model. In this strain, mutations in the *Tyrp1* and *Gpnmb* genes, which encode tyrosinase-related protein 1 and transmembrane glycoprotein Nmb, play a crucial role (Anderson et al., 2002). These mutations are thought to initiate the demise of iris cells by inducing toxicity related to melanogenesis. This leads to blocked outflow of aqueous fluid, resulting in increased IOP and canonical glaucoma symptoms (Libby et al., 2005).

The IOP independent primary genetic glaucoma model reassembles normal tension glaucoma (NTG) and involves genetic mutation within *Tbk1* and *Glast* genes (Harada et al., 2007, 2010; Fingert et al., 2017). Mutations in the *Tbk1* gene have been linked to NTG, a form of glaucoma that manifests with low or average IOP. Multiplications of the *Tbk1* gene have been identified in approximately 1% of NTG cases, affecting individuals from various ethnic backgrounds. The precise mechanism through which mutations in *Tbk1* gene contribute to NTG remains unclear. Nevertheless, this gene encodes proteins that play roles in the NF-κB signaling pathway and autophagy. Fingert et al. (2017) generated a transgenic mouse strain (referred to as Tg-TBK1) by integrating a single wild-type copy of the human *TBK1* gene, along with its native promoter, into the mouse genome. Their findings demonstrated that these mice experience progressive retinal ganglion cell (RGC) loss despite maintaining normal IOP values. Within the mammalian retina, the glutamate/aspartate transporter (GLAST) plays a crucial role as a primary glutamate transporter. Depletion of GLAST is associated with optic nerve degeneration, resembling the characteristics of NTG, which can be explained by impaired oxidative balance due to low content of glutathione and subsequent excitotoxic insult (Harada et al., 2007, 2010).

Secondary glaucoma models with manual induction of increased IOP can be based among others on laser photocoagulation of the aqueous outflow tract, different microbeads obstruction of trabecular meshwork, as well as injection of hypertonic saline into the episcleral veins. These techniques result in a moderate increase in IOP, resulting in progressive RGCs loss and optic nerve degeneration (Morrison et al., 1997; Grozdanic et al., 2003; Moreno et al., 2005; Smedowski et al., 2014; Kimura et al., 2020; Pang and Clark, 2020). These techniques have several advantages and disadvantages, for example, the laser technique requires expensive, specialized equipment, while modulation of laser intensity and duration offers the possibility of great control over the induction of IOP. Saline injection requires less equipment, but it is a surgical technique that requires a great deal of precision and training, due to the small size of rodent ocular vessels. Microbeads injection is relatively simple and reproducible method; however, it may obscure the visual axis of the eye, making functional measurements impossible.

The secondary, inducible IOP-independent glaucoma models represent intraocular injection of excitotoxic agents into the vitreous body, which induce excessive activation of ionotropic glutamate receptors, resulting in an increase in intracellular calcium levels and ultimately to cell damage and death (Pang and Clark, 2020). Cells such as horizontal cells, bipolar cells, ganglion cells, and amacrine cells possess three types of ligand-gated ion channels: N-methyl-D-aspartate (NMDA) receptors, α-amino-3-hydroxy-5-methyl-4-isoxazolopropionic acid (AMPA) receptors, and kainate receptors. The targeted neurodegeneration of cells in the inner layers of the retina, including RGCs, is induced by the intraocular administration of ligands for these receptors (Sun et al., 2001; Casson and Franzco, 2006; Christensen et al., 2019).

Currently, most studies utilizing animal glaucoma models rely on quantification of RGCs to track the progression of neurodegeneration. There are many methods to visualize RGCs in wild-type and genetically engineered retinas, such as immunofluorescence staining with RGCs-specific antibodies (Brn3a, RBPMS, Tuj1, Thy1.1, Islet1; Nadal-Nicolás et al., 2009; Smedowski et al., 2014, 2016, 2018a; Pietrucha-Dutczak et al., 2017; Pacwa et al., 2023) or the use of retrograde tracers, such as FluoroGold (Smedowski et al., 2018b; Hu et al., 2021). Most of the animal models currently used show a rapid rate of degeneration of RGCs, which is atypical for most of human chronic glaucoma cases, but, up to now, these models are the most reliable tools for studying the mechanisms of RGCs degeneration and finding new drug targets.

## Molecular mechanisms of glaucoma development

### TGF-β signaling

Transforming growth factor beta (TGF-β) is a protein belonging to a larger family called the transforming growth factor beta superfamily, which includes bone morphogenetic proteins (BMPs), inhibins, activins, differentiation growth factors and other signaling molecules (Shi and Massagué, 2003; Massagué, 2012). TGF-β is a cytokine involved in signaling cascades related to cell differentiation, cell division, chemotaxis, and fibrosis. TGF-β has three isoforms: TGF-β1, TGF-β2, TGF-β3, of which TGF-β2 is most associated with eye function (De Caestecker, 2004). The cellular effect of TGF-β signaling depends on the type of protein isoform and its tissue localization. One of the effects of TGF-β is increased extracellular matrix production and remodeling.

**Figure 2:**
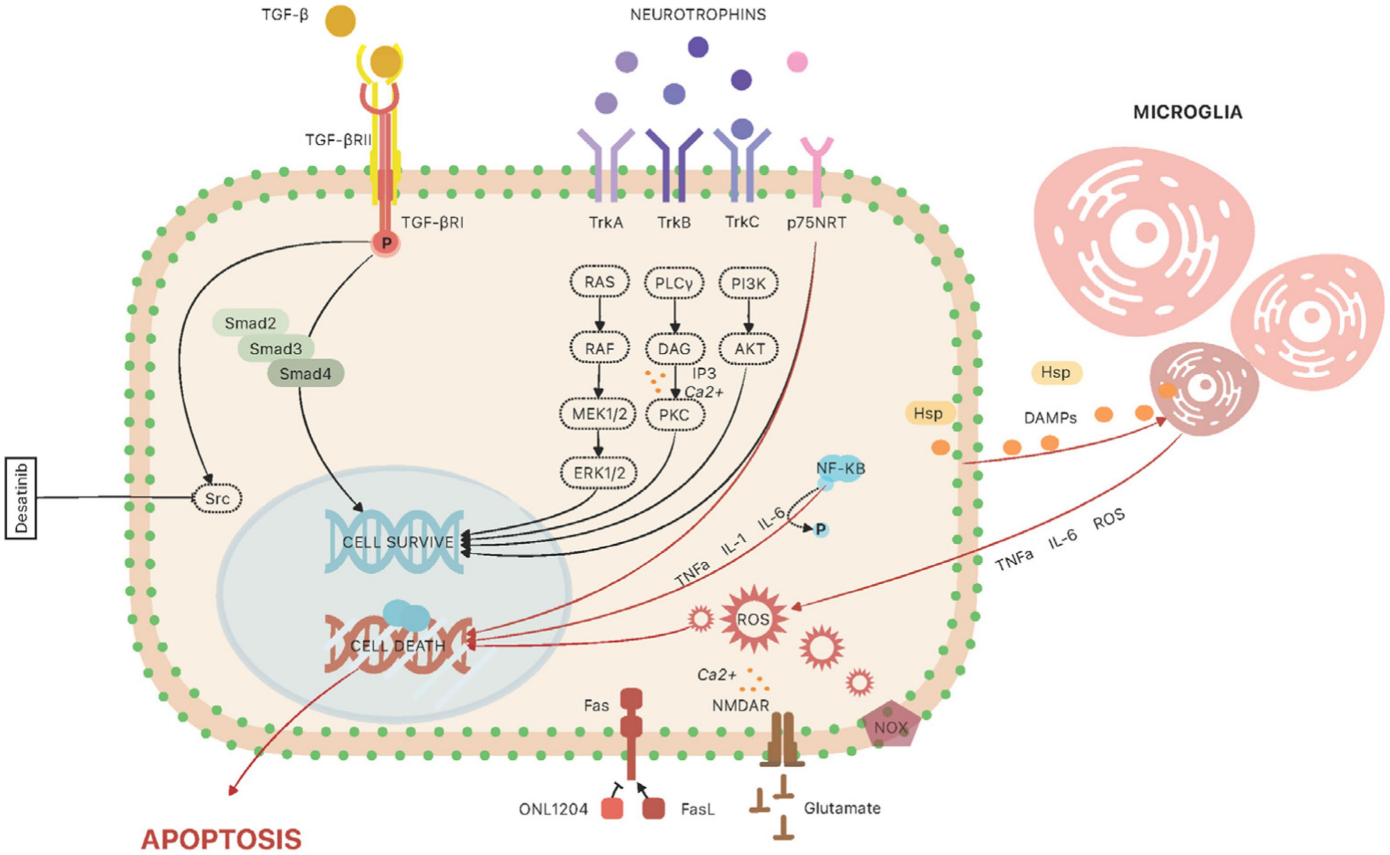
The molecular pathways involved in animal glaucoma models at the level of retinal ganglion cell. *TGF-β signaling*: The canonical TGF-β signaling pathway begins with its binding to the type II receptor (TGF-βRII), which leads to phosphorylation and activation of the type I receptor (TGF-βRI). This process activates intracellular SMADs proteins (Smad2, Smad3, Smad4). The Smad complex moves to the cell nucleus, where it activates gene transcription, leading to the production of extracellular matrix. In trabecular meshwork cells, studies have shown that another important regulator, involved in TGF-β2-induced IOP increase, is Src kinase; the use of a potent Src inhibitor, Desatinib, resulted in alleviation of TGF-β2-induced IOP increase in rat eyes. *Neurotrophins*: The neurotrophic pathways provide support and protection to RGCs, and the loss of this support can contribute to the death of RGCs in glaucoma. The action of neurotrophins is mainly manifested through activation of two types of transmembrane glycoproteins, tropomyosin receptor kinases (TrkA, TrkB, and TrkC) and the low affinity neurotrophin receptor p75NTR. Trk activation triggers intricate signaling cascades, activating the Ras/ERK (extracellular signal-regulated kinase) pathway and stimulating mitogen-activated protein kinases (MAPs). Simultaneously, the PI-3 kinase/Akt pathway engages, and phospholipase C (PLC)-γ1 is activated (Mallone et al., 2020). The activation of p75NTR arises from the binding of immature precursor forms of neurotrophins (proNT), eliciting a dual effect on cell fate—either apoptosis or survival. This outcome is contingent upon the simultaneous expression of Trk receptors. Consequently, maintaining cellular homeostasis hinges on achieving equilibrium in the availability of proNTs and their mature counterparts, alongside the presence of both Trk and p75NTR receptors. *Neuroinflammation*: Microglia and astroglia are the cells involved in inflammatory mechanisms in the retina. Their function is to regulate ion concentration, metabolic support of neurons and neuroprotective activity. Microglia cells, once mature, can be activated by damage-associated molecular pathways (DAMPs), which are released by neuronal cells. In addition, increased IOP stimulates RGCs to produce and secrete heat shock proteins (HSPs). These factors stimulate microglial cells to secrete cytokines and chemokines, such as tumor necrosis factor-alpha (TNF-α), interleukin-6 (IL-6) and complement factors, which promote the morphological transformation of microglia into M1 (proinflammatory) and M2 (anti-inflammatory) macrophages. Inflammatory pathways are involved in the regulation of various genes that are upregulated in the retina. One of the regulatory factors is nuclear factor-kappa B (NF-κB)—it leads to upregulation of IL-1 cytokine family, IL-6 and TNF-α. TNF-α and another proapoptotic protein, FasL, have been implicated in the pathogenesis of glaucoma. *Fas/FasL signaling*: Fas ligand (FasL) is a protein that belongs to the TNF family. When bound to the Fas receptor, it causes induction of apoptotic and pro-inflammatory pathways. This pathway has been identified as involved in axonal degeneration, death of RGCs and induction of neuroinflammation in a chronic and induced mouse model of glaucoma. Another study used the small peptide molecule ONL1204, an antagonist of the Fas receptor, which was administered to mice by injection into the vitreous body. *NADPH oxidase*: Several recent studies have pointed to a significant role for NADPH oxidase (NOX) in oxidative stress during glaucoma. NOX has been identified as a major source of oxidative stress in many retinal eye diseases, such as age-related macular degeneration and ischemic retinopathy. Reactive oxygen species produced by NOX affect the regulation of various cellular processes such as proliferation, differentiation, and migration. *Excitotoxicity*: Glutamate plays an important function in regulating neurophysiological processes. However, increased stimulation of relevant receptors with glutamate leads to neuronal damage and death.

The canonical TGF-β signaling pathway begins with its binding to the type II receptor, which leads to phosphorylation and activation of the type I receptor. This process activates intracellular SMADs proteins (Smad2, Smad3, Smad4), which form an oligomeric complex with co-SMAD (Feng and Derynck, 2005; Massagué et al., 2005). The Smad complex moves to the cell nucleus, where it activates gene transcription, leading to the production of extracellular matrix. In the healthy eye, TGF-β2 is involved in corneal healing and scar formation (De Caestecker, 2004). Studies in mice have shown that overexpression of TGF-β2 induces an IOP elevation in wild-type mice, while this effect was not observed in Smad3 knockout mice – thus inferring the importance of Smad proteins in the molecular mechanisms of glaucoma development (McDowell et al., 2013; Figure 2).

Studies have shown that another important regulator, involved in TGF-β2-induced IOP increase, is Src kinase. The application of a potent Src inhibitor, Dasatinib, led to the mitigation of TGF-β2-induced IOP elevation in rat eyes (Tsukamoto et al., 2019). Moreover, investigations conducted on human trabecular meshwork cells demonstrated that TGF-β2 activates Src signaling, promoting enhanced cell adhesion, cytoskeletal remodeling, and the accumulation of extracellular matrix (ECM). Furthermore, the use of a Src inhibitor attenuated TGF-β2-induced changes. Src activity plays an important role in the TGF-β-induced elevation of intraocular pressure (IOP). Identifying Src signaling as a potential therapeutic target in glaucoma suggests promising avenues for intervention.

### Neurotrophins

Neurotrophins (NTs) are central regulators, exerting a critical influence on diverse signaling pathways essential for both neuronal survival and differentiation. Additionally, they play a significant role in promoting synaptic plasticity and facilitating axon regeneration (Rudzinski et al., 2004; Skaper, 2008; Mallone et al., 2020). The neurotrophic pathways are also involved in the development of glaucoma (Rudzinski et al., 2004; Johnson et al., 2011; Weissmiller and Wu, 2012). The neurotrophic pathways provide support and protection to RGCs, and the loss of this support can contribute to the death of RGCs in glaucoma (Guo et al., 2014; Nuschke et al., 2015; Oddone et al., 2017; Dias et al., 2022; Figure 2). Recent literature highlights that heightened intraocular pressure (IOP) and oxidative stress are implicated in the reduction of available neurotrophic factors, ultimately contributing to the demise of retinal ganglion cells (RGCs) in glaucoma. The neurotrophin family comprises pivotal members such as nerve growth factor (NGF), brain-derived neurotrophic factor (BDNF), neurotrophin 3 (NT3), and neurotrophin 4/5 (NT4/5). Neurotrophin activities primarily hinge on the activation of two distinct transmembrane glycoproteins: tropomyosin receptor kinases (TrkA, TrkB, and TrkC) and the low-affinity neurotrophin receptor p75NTR. Trk activation triggers intricate signaling cascades, activating the Ras/ERK (extracellular signal-regulated kinase) pathway and stimulating mitogen-activated protein kinases (MAPs). Simultaneously, the PI-3 kinase/Akt pathway engages, and phospholipase C (PLC)-γ1 is activated. The activation of p75NTR arises from the binding of immature precursor forms of neurotrophins (proNT), eliciting a dual effect on cell fate-either apoptosis or survival. This outcome is contingent upon the simultaneous expression of Trk receptors. Consequently, maintaining cellular homeostasis hinges on achieving equilibrium in the availability of proNTs and their mature counterparts, alongside the presence of both Trk and p75NTR receptors (Levi-Montalcini et al., 1996; Frade and Barde, 1998; Skaper, 2008; Al-Qudah and Al-Dwairi, 2016; Platholi and Lee, 2018).

Nerve growth factor (NGF), the inaugural and extensively studied member of the neurotrophins (NTs) family, plays a pivotal role in neuronal differentiation and survival. Numerous studies highlight the influence of NGF on these processes, particularly within the optic nerve and visual pathway. Expression of NGF is observed in key ocular structures such as the retinal pigment epithelium, Müller cells, and retinal ganglion cells. Correspondingly, NGF receptors are expressed in these structures as well as in photoreceptors (Chakrabarti et al., 1990). Experimental investigations underscore the significance of NGF in promoting the survival and development of RGCs through its role as a local paracrine and autocrine mediator (Colafrancesco et al., 2011; Allen et al., 2013; Garcia et al., 2014; Roberti et al., 2014; Wang et al., 2014; Aloe et al., 2015; Chen Q. et al., 2015; Mallone et al., 2020; Li et al., 2022; Lin et al., 2022). NGF is delivered retrogradely along the axons of RGCs (Dias et al., 2022). Sposato et al. (2009) treated rats having elevated IOP with topical application of NGF. The results showed that increased IOP alters the basal content of NGF and NGF receptors in visual centers in brain and that NGF topical application normalized these deficits. These findings suggest that degeneration in retinal areas may contribute to a reduction in NGF levels. Following above, the clinical trial of topical application of NGF in glaucoma has been initiated (Beykin et al., 2022).

Another of NTs is brain-derived neurotrophic factor (BDNF), which, due to its potential neuroprotective effects, is the subject of much research in developing therapies for diseases such as Parkinson’s disease, amyotrophic lateral sclerosis, Alzheimer’s disease, and glaucoma (Arancibia et al., 2008; Greenberg et al., 2009; Allen et al., 2013; Domenici et al., 2014; Kimura et al., 2016). Synthesized in the brain, BDNF undergoes retrograde transport to RGCs (Dias et al., 2022). Impaired axonal transport from the brain to RGCs is considered a critical factor contributing to RGC death and optic nerve degeneration in glaucoma. BDNF is also locally produced in the ganglion cell layer (GCL), the inner nuclear layer, and in Müller cells. Notably, retinal BDNF levels were observed to increase in a pressure-induced animal model of glaucoma (Guo et al., 2010). Increased retinal BDNF level was shown to be neuroprotective in optic nerve injury models, including glaucoma (Ritch and Sharma, 2000; Arancibia et al., 2008; Domenici et al., 2014; Sanna et al., 2017; Wójcik-Gryciuk et al., 2020). BDNF plays a crucial role in maintaining the integrity of the inner retina under normal conditions, and its deficiency has been linked to retinal and optic nerve damage during progressive glaucoma (Gupta et al., 2014). The functional activity of BDNF is manifested through its interaction with the TrkB receptor, leading to the activation of intracellular signaling pathways associated with cell viability, including the phosphatidylinositol-3 kinase (PI-3 K)/Akt and extracellular signal-regulated kinases 1/2 (Erk1/2) pathways. Both of these signaling pathways have been implicated in experimental glaucoma (Cheng et al., 2002; Carlotta et al., 2016; Liang et al., 2019). There are several studies using different models of glaucoma that indicate that local administration of BDNF to the eye results in reduced loss of RGCs. Moreover, there is speculation that combining BDNF administration to the eyeball with administration to visual centers in the brain could enhance the survival of RGCs in animals with induced optic nerve damage (Weber et al., 2010; Weber and Harman, 2013).

### Neuroinflammation

Several studies suggest that glaucoma development has analogies with neurodegenerative diseases connected with inflammatory responses (Yang et al., 2011; Olmos and Lladó, 2014; Wilson et al., 2015; Parsadaniantz et al., 2020; Rolle et al., 2021; Wong and Benowitz, 2022). In this chapter, we will discuss the possible molecular mechanisms of neuroinflammation development (Figure 2).

Within the retina, microglia and astroglia emerge as pivotal players in the orchestration of inflammatory processes (García-Bermúdez et al., 2021; Quan et al., 2022). Their function is to regulate ion concentration, metabolic support of neurons and neuroprotective activity. Microglia cells, once mature, can be activated by damage-associated molecular pathways (DAMPs), which are released by neuronal cells (Gülke et al., 2018). In addition, increased IOP stimulates RGCs to produce and secrete heat shock proteins (HSPs; Piri et al., 2016). These factors stimulate microglial cells to secrete cytokines and chemokines, such as tumor necrosis factor-alpha (TNF-α), interleukin-6 (IL-6) and complement factors, which promote the morphological transformation of microglia into M1 (proinflammatory) and M2 (anti-inflammatory) macrophages (Li and Barres, 2018). Microglial cells communicate with astroglia by secreting signaling molecules that can lead to the differentiation of astroglia cells into two phenotypes: harmful A1 and neuroprotective A2 (Li et al., 2020). Both of these cell types, microglia and astroglia, are significantly involved in the formation and regulation of neuroinflammation.

Inflammatory pathways are involved in the regulation of various genes that are upregulated in the retina. One of the regulatory factors is nuclear factor-kappa B (NF-κB) – it leads to upregulation of IL-1 cytokine family, IL-6 and TNF-α. TNF-α and another proapoptotic protein, FasL, have been implicated in the pathogenesis of glaucoma (Gregory et al., 2011). Recent research has focused on identifying new therapeutic targets that can reduce or completely abolish the deleterious effects of neuroinflammation.

Astroglial NF-κB has been identified as one of the possible therapeutic targets for modulating the inflammatory response (Yang et al., 2020). The experimental use of mice with induced glaucoma and with or without deletion of astroglial IκKβ, which is the main activating kinase involved in IκB degradation through the canonical pathway of NF-κB activation, showed reduced production of inflammatory cytokines in brain ganglia lacking IκKβ, including TNF-α, which can induce apoptosis of RGCs and axonal degeneration in glaucoma.

Another target of research is Fas/FasL signaling pathway. Fas ligand (FasL) is a protein that belongs to the TNF family. When bound to the Fas receptor, it causes induction of apoptotic and pro-inflammatory pathways (Ju et al., 1995; Gregory et al., 2011). This pathway has been identified as involved in axonal degeneration, death of RGCs and induction of neuroinflammation in a chronic and induced mouse model of glaucoma (Krishnan et al., 2016, 2019) Another study used the small peptide molecule ONL1204, an antagonist of the Fas receptor, which was administered to mice by injection into the vitreous body (Krishnan et al., 2019). The results showed reduced death ratio of RGCs in high-tension glaucoma model. Reduced expression of genes involved in the pathogenesis of glaucoma was also observed, including chemokines and cytokines (GFAP, Caspase-8, TNFα, IL-1β, IL-6, IL-18, MIP-1α, MIP-1β, MIP-2, MCPI, and IP10), components of the complement cascade (C3, C1Q), Toll-like receptor pathway (TLR4).

Recent studies in animal models suggest that monocyte-like cells enter the optic nerve head in an ocular hypertensive mouse model of glaucoma (DBA/2 J) and produce damaging molecules that lead to neurodegeneration (Howell et al., 2012; Wilson et al., 2015; Williams et al., 2017). However, the mechanisms of this action are not fully understood. To study these interactions, monocyte infiltration inhibitors were used: DS-SILY, a peptidoglycan that provides a barrier to platelet adhesion to the vessel endothelium and to monocytes, and genetic targeting of *Itgam* (CD11b, an immune cell receptor that blocks extravasation of immune cells; Williams et al., 2019). The results identified new monocyte-related inflammatory pathways and blocking these pathways reduced monocyte infiltration and provided neuroprotection in DBA/2 J mice.

The anti-inflammatory effects of Ibudilast, a cAMP phosphodiesterase inhibitor, were also investigated in a rat model of ocular hypertension (Vargas et al., 2016). Intraocular administration of Ibudilast reduced macroglia and microglia reactivity in the retina and optic nerve, thereby decreasing the amount of pro-inflammatory cytokines secreted. The study also showed that elevated IOP increased A-type PDE4 activity in Müller cells, and Ibudilast administration resulted in an increase in cellular cAMP. The combined injection of Ibudilast and Rp-cAMP (a non-hydrolysable analog of cAMP that inhibits protein kinase A) resulted in complete blockade of Ibudilast-induced neuroprotection. These results indicate that Ibudilast may be a well-tolerated and safe modulator of neuroinflammation in glaucoma, and that PDE4 represents a potential target for research into inhibiting chronic gliosis and neuroinflammation in glaucoma (Rolle et al., 2021).

Another pathway involved in neuroinflammation represents Toll-like receptors (TLR) signaling, particularly TLR2 and TLR4 present in microglia. Studies have shown that these pathways are involved in the transmission of signals induced by heat shock proteins and are associated with oxidative stress (Luo et al., 2010). It has also been observed that Tenascin C, which is one of the ligands for TLR4, undergoes increased expression both in human glaucoma patients and in animal models (Johnson et al., 2007; Wallace et al., 2015). TLRs are therefore an interesting target for research on inhibition and reduction of neuroinflammation. There are studies intensively using human umbilical cord mesenchymal stem cells (hUC-MSCs) to show their immunomodulatory properties (Cislo-Pakuluk and Marycz, 2017). These cells show the ability to regulate the function of microglia and astrocytes (Jose et al., 2014; Wang et al., 2018). They have also been used in studies to inhibit the activity of TLRs, with a positive effect in reduced neuroinflammation in a rat model of glaucoma (Lund et al., 2009; Zwart et al., 2009; Ji et al., 2018).

The NLRP3 inflammasome, a complex of multiple proteins, plays a crucial role in initiating inflammatory responses. Comprising NLRP3, ASC (apoptosis-associated speck-like protein containing a caspase recruitment domain), and pro-caspase-1, this inflammasome is activated by various triggers. Upon activation, pro-caspase-1 undergoes cleavage into its active form, initiating the maturation and release of pro-inflammatory cytokines, including interleukin-1β (IL-1β) and interleukin-18 (IL-18; Chi et al., 2014).

Recent research has compellingly demonstrated the activation of the NLRP3 pathway in different types of glaucoma. Elevated levels of NLRP3 inflammasome components have been identified in the trabecular meshwork, retina, and optic nerve head of eyes affected by glaucoma (Ward et al., 2019). This upregulation of NLRP3 is closely associated with neuroinflammation and the death of retinal ganglion cells (RGCs), contributing to the progressive nature of glaucomatous optic neuropathy. A noteworthy connection has been suggested between the activation of the NLRP3 inflammasome and increased intraocular pressure (IOP). Mechanical stress, a common factor in glaucoma, may serve as a trigger for NLRP3 inflammasome activation, resulting in the release of pro-inflammatory cytokines. This cascade of events is implicated in the perpetuation of neuroinflammation and the subsequent damage to the optic nerve. The potential therapeutic implications of targeting the NLRP3 pathway in glaucoma are promising. Inhibiting NLRP3 activation, whether through pharmacological agents or gene therapies, has demonstrated neuroprotective effects in experimental models (Coll et al., 2015; Gong et al., 2019; Zhang et al., 2019; Coyle et al., 2021). However, further research is necessary to thoroughly explore the safety and efficacy of these interventions, paving the way for potential translation into clinical applications.

### Chronic oxidative stress

Oxidative stress is an imbalance between free radicals and antioxidant scavengers that can result in tissue damage (Pickering et al., 2013). Reactive oxygen species (ROS) are free radicals that are the main source of oxidative stress formation. These include superoxide anion (O_2_^−^), lipid radical, hydroxyl radical and nitric oxide (NO). Uncontrolled production of ROS leads to oxidation of biological molecules such as proteins, lipids and DNA contained in the cell, which triggers activation of mechanisms leading to cell death by apoptosis or necrosis (Garza-Lombó et al., 2020). There are several mechanisms leading to ROS production, and the most important include uncoupled NO synthase, mitochondrial dysfunction, xanthine oxidase and NADPH oxidase (Dalle-Donne et al., 2006). Mechanisms related to oxidative stress are considered an important factor in the development of neurodegenerative diseases, including glaucoma (Levkovitch-Verbin et al., 2000; Himori et al., 2013; Lin and Kuang, 2014; Naguib et al., 2021; Sanz-Morello et al., 2021).

Autophagy is a lysosomal degradation of the cellular constituents. Cellular homeostasis is maintained by removing damaged organelles, such as mitochondria, in the autophagic process of mitophagy (Terman et al., 2006). However, intralysosomal accumulation of waste material increases with age. Oxidation of this material leads to the formation of a non-degradable substance called lipofuscin (Grune et al., 2005). Lipofuscin has been shown to accumulate in glaucomatous human lamina cribrosa cells. This accumulation may in turn alter cellular authophagic activity. In addition, autophagy markers have been found in lamina cribrosa cells (McElnea et al., 2011). Furthermore, studies using DBA/2 J mice showed that in myelinated optic nerve axons the number of mitochondria was increased, and they had significantly smaller surface area. These results indicate that mitochondrial pathology may be the cause of the energy deficit in glaucomatous optic nerve. An increased number of autophagosomes was observed, which, together with increased number of mitochondria and decreased *Lamp1* (lysosomal associated membrane protein 1) gene, suggests that deteriorating mitochondria do not recycle efficiently enough in the mitophagy process (Coughlin et al., 2015). These findings suggest that mitochondrial dysfunction and the resulting oxidative stress may be an important factor in the pathogenesis of glaucoma.

In addition to the well-known correlation between mitochondrial dysfunction and oxidative stress, several recent studies have pointed to a significant role for NADPH oxidase (NOX) in oxidative stress during glaucoma. NOX has been identified as a major source of oxidative stress in many retinal eye diseases, such as age-related macular degeneration and ischemic retinopathy (Ruan et al., 2020). Reactive oxygen species produced by NOX affect the regulation of various cellular processes such as proliferation, differentiation, and migration (Chan et al., 2009). There are seven NOX isoforms, three of which are the most studied in the eye pathology: NOX1, NOX2 and NOX4. NOX1 and NOX2 produce superoxide, while NOX4 mainly produce hydrogen superoxide (Griendling et al., 2000). The enzymatic activity of NOX can be induced by various stimuli: hypoxia (Dvoriantchikova et al., 2012), proinflammatory cytokines, i.e., TNF-α (Geng et al., 2020) or heat shock proteins (Sakai et al., 2003). All of these stimuli are associated with glaucoma pathology. There are many studies using animal glaucoma models that show the relationship between NOX activity and pathological features (Yokota et al., 2011; Dvoriantchikova et al., 2012). Recently, many studies have focused on using pharmacological inhibition to block the contribution of NADPH oxidase in an animal model of retinal pathological conditions, such as ischemic retinopathy and retinal inflammation. However, there are very limited studies on the pharmacological inhibition of NOX in animal glaucoma models. In the ischemia reperfusion (I/R) injury model, the role of NOX2 in neuronal cell death was also examined (Yokota et al., 2011). The results show that I/R induces the retinal cell death, indicated by numerous TUNEL-positive cells in the inner retina and significant decrease in neuronal cell density. This effect was abolished in mice with *Nox2* gene deletion, indicating a role for NOX2 NADPH oxidase in I/R-induces injury of the inner retina. Furthermore, Apocynin, a non-selective inhibitor of NADPH oxidase, was used in the study. Apocynin improved retinal morphology in NOX2^−/−^ with I/R injury, indicating the need for studies using other selective NOX inhibitors in glaucoma models.

### Excitotoxicity

Glutamate plays an important function in regulating neurophysiological processes. However, increased stimulation of relevant receptors with glutamate leads to neuronal damage and death. A similar mechanism takes place in the eye. Glutamate is secreted by ganglion cells, bipolar cells, and photoreceptors in response to light stimuli. Its homeostasis is ensured by the activity of microglia and macroglia cells. When glutamate homeostasis is disrupted, its uncontrolled increased concentration leads to excitotoxicity (Harada et al., 2007). Glutamate binds to the N-methyl-D-aspartic acid receptor (NMDAR), affecting the disruption of K^+^/Na^+^ ion homeostasis—there is an increased influx of Na^+^ ions into cells, resulting in the release of calcium ions, activation of the ERK 1/2 signaling pathway, formation of reactive oxygen species (ROS), oxidative stress and mitochondrial dysfunction (Arundine and Tymianski, 2003). All these factors lead to apoptotic death of retinal cells. Intravitreal injection of glutamate in rats resulted in loss of RGCs, which is considered a major cause of vision loss in glaucoma (Lam et al., 1999).

There are attempts to interfere with the mechanisms of excitotoxicity in ischemic models. The first idea was the use NMDA receptor antagonists in a rat model of retinal ischemia, but its complete blockade affects other physiological processes of nerve cells (Gómez-Vicente et al., 2015). Another approach was the use of brimonidine, a selective α_2_-adrenergic agonist that shows the ability to lower IOP and prevent the excitotoxicity in RGCs. Its proven mechanism of action is based on lowering glutamate secretion, regulating Ca^2+^ ion homeostasis, reducing NMDA receptor expression, or decreasing production of aqueous humor by the ciliary body (Lee et al., 2012; Villanueva et al., 2020). *In vivo* studies showed that intrathecal injection of brimonidine prevented the death of RCGs in a rabbit model with NMDA-induced excitotoxicity and in rat model of high-tension glaucoma (Dong et al., 2008; Ozdemir et al., 2009).

Other studies use glutamate transporters found in retinal cells as targets. One of these, called GLAST, found mainly in Müller cells, removes most glutamate molecules from the extracellular space (Vorwerk et al., 1999). Studies using GLAST knock-out mice have shown that about 50% of glutamate is captured by GLAST (Shi et al., 1997). Other studies indicate that increased IOP in animal models results in decreased GLAST expression (Schuettauf et al., 2007) and knocking out *Glast* gene leads to spontaneous degeneration of RGCs in normal tension conditions (Harada et al., 2010). Increasing glutamate transport is therefore an interesting target for studies on reducing pressure-induced neurodegeneration. There are studies in which the estrogen modulator, Tamoxifen, was used and showed neuroprotective properties due to up-regulation of GLAST, which reduced excitotoxicity (Lee et al., 2009). In a rat model of glaucoma, eye drops containing 17β-Estradiol were also used, which had the effect of reducing neurodegeneration (Prokai-Tatrai et al., 2013). Estradiol and its analogs are therefore interesting targets capable of increasing GLAST expression, which could be exploited in glaucoma patients.

### ABCA1

Recent research has linked ATP binding cassette transporter A1 (ABCA1) to POAG. ABCA1 is a member of a superfamily of ABC transmembrane transporters responsible for removing cholesterol by transferring it to lipid-free apolipoprotein A-I and apolipoprotein E (Danik et al., 1999). Its transcription is regulated by liver X receptor (LXR), a member of the nuclear receptors superfamily (Yang et al., 2011). ABCA1 is prominently expressed in various ocular tissues including the trabecular meshwork, Schlemm’s canal, endothelial cells, optic nerve, and retina, particularly in retinal ganglion cells (Cai et al., 2015). Recent studies suggest ABCA1’s potential role in regulating intraocular pressure (IOP), although the precise mechanism remains unclear. Hu et al. propose a connection between ABCA1-mediated IOP regulation and the Cav1/eNOS/NO signaling pathway, which is implicated in glaucoma pathogenesis in POAG patients. ABCA1 interacts with caveolin-1 (Cav1), a key player in cellular processes like vesicular transport, cholesterol regulation, and mechanosignal transmission (Kuo et al., 2011; Gu et al., 2017). Cav1 regulates vasodilation by modulating endothelial nitric oxide synthase (eNOS), known as a pressure-dependent regulator of IOP through the conventional outflow tract pathway (Daniel Stamer et al., 2011). ABCA1 expression levels were examined in samples of trabecular meshwork from POAG patients and human donor eyes. ABCA1 expression levels were analyzed in samples of trabecular meshwork from POAG patients and human donor eyes, showing higher expression in POAG patients compared to controls. Studies on cells and animal models suggest that ABCA1 can modulate Cav1 expression, alleviating Cav1’s inhibitory effect on eNOS, thus increasing eNOS-derived production and reducing IOP (Hu et al., 2020). Based on these findings, enhancing the ABCA1 signaling pathway is proposed as a potential therapeutic approach for glaucoma and ocular hypertension treatment.

### Rho-kinase

The Rho kinase (ROCK) activation pathway is a critical signaling pathway involved in various cellular processes such as cell migration, proliferation, adhesion, and smooth muscle contraction (Thumkeo et al., 2013). Rho-associated protein kinases (ROCK) are a serine/threonine protein kinases, that are downstream effectors of small GTPase Rho. The activation process involves the binding of Rho-GTP to the kinase domain of ROCK, resulting in a conformational change that enhances ROCK activity. Activated ROCK phosphorylates various downstream substrates, including myosin light chain (MLC), myosin phosphatase substrate 1 (MYPT1), kinase C-potentiated phosphatase inhibitor 17 (CP1-17), LIM kinase (LIMK), calmodulin (CaM), ezrin, radixin, and moesin. Through these interactions, the Rho-ROCK signaling pathway orchestrates the dynamics of actin cytoskeleton, contraction mediated by actomyosin, adherence between cells, cellular morphology, and the reorganization of the extracellular matrix (ECM; Amano et al., 2010). Researchers have indicated that ROCK inhibitors have the capability to influence trabecular meshwork, enhancing the drainage of aqueous humor from the eye and subsequently decreasing IOP (Honjo and Tanihara, 2018). Currently, there are several ROCK inhibitors in use as a novel class of drugs that can act directly on trabecular meshwork. Three of them (ripasudil, netarsudil and fasudil) are commercially used in the treatment of glaucoma in some countries. Two of the ROCK inhibitors have not been approved yet (SNJ-1656—phase II clinical trial, Y-27632—pre-clinical phase; Pagano et al., 2023). With the rise in the use of ROCK inhibitors by ophthalmologists, it becomes important to enhance our comprehension of the potential advantages and safety profiles associated with these pharmaceuticals, whether employed in approved or off-label capacities.

### Purinergic signaling pathway

Purinergic P2 receptors binds extracellular nucleotides. The superfamily contains two types of receptors. First, G protein-coupled P2Y binds ATP, ADP, UTP, UDP, UDP-sugars and interacts with G proteins inducting intracellular signaling pathways, and ionotropic P2X, which binds ATP and has two or three transmembrane domains and form an ion channel gating Na^+^, K^+^, and Ca^2+^ (Shinozaki et al., 2023). Normally, the level of extracellular ATP is extremely low. ATP levels can rise due to uncontrolled release from necrotic cells or controlled release via exocytosis or channel-mediated mechanisms. Ocular tissues like the lens, retina, corneal endothelial cells, ciliary body, and retinal astrocytes are known to release ATP and nucleotides. There are indications that a sudden increase in intraocular pressure (IOP) prompts ATP release from the retina. Within the dynamics of aqueous humor, an increase in ATP or UTP prompts the activation of P2Y_2_ receptors, resulting in an escalation of aqueous humor production. On the other hand, when there’s less ADP/UDP, it stops the receptors P2Y_1/6_ from slowing down or speeding up fluid production or drainage (Shevozaki et al., 2005; Taguchi et al., 2016; Hamada et al., 2021). These changes make the IOP go up, which could harm the RGCs. Disrupted purinergic signaling may damage RGCs through autonomous and non-autonomous pathways. P2X7 receptors, found in RGCs, microglia, astrocytes, and Müller cells, play an important role. RGCs experience excitotoxicity when these receptors are activated. In glial cells, activation leads to neurotoxic responses, including microglia releasing pro-inflammatory cytokines and ROS that harm RGCs. Astrocytes release glutamate upon activation, overstimulating glutamate receptors in RGCs, while Müller cells reduce glutamate uptake, further damaging RGCs. Additionally, P2X_7_ receptor activation in Müller cells triggers inflammation.

Shevozaki et al. (2005) showed that P2Y_6_ lowers IOP – ablation of the P2Y_6_ gene in mice (P2Y6 KO) results in hypertensive glaucoma–like optic neuropathy—exhibited sustained elevation of IOP, age-dependent damage to the optic nerve, thinning of ganglion cell inner layers, and a reduction of RGC numbers. Topically applied uridine diphosphate, an endogenous selective agonist for the P2Y_6_ receptor, decreases IOP. In that case P2 receptor ligands may be a promising candidate for glaucoma treatment.

## Conclusion

Animal glaucoma models are used to study various molecular pathways associated with the development and progression of glaucoma, which is a complex eye disease characterized by progressive vision loss, involves the dysregulation of numerous molecular pathways. These pathways encompass various mechanisms critical to the pathogenesis of the condition. Among them are processes associated with intraocular pressure (IOP) regulation, neuroinflammation, oxidative stress, and neurodegeneration, which contribute to the damage of retinal ganglion cells and their axons. Excitotoxicity, vascular dysregulation, and gliosis further exacerbate neuronal injury, while deficits in axonal transport and protein misfolding/aggregation disrupt cellular function and integrity. Additionally, abnormalities in autophagy and apoptosis pathways contribute to tissue degeneration. However, the exploration of neuroprotective mechanisms and the influence of genetic factors offer hope for understanding and potentially mitigating the progression of glaucoma. By studying these molecular pathways in animal glaucoma models, researchers aim to gain insights into the disease’s mechanisms and identify potential therapeutic targets for the treatment and prevention of glaucoma. By understanding the molecular mechanisms of glaucoma, we can develop new strategies to prevent or slow the progression of this debilitating disease and preserve vision.

## Author contributions

KB: Conceptualization, Software, Writing – original draft, Writing – review & editing. AP: Funding acquisition, Software, Writing – original draft, Writing – review & editing. AS: Conceptualization, Funding acquisition, Resources, Supervision, Writing – original draft, Writing – review & editing.
